# Clinical, myopathological, and genetic features of two Chinese families with Andersen-Tawil syndrome

**DOI:** 10.3389/fneur.2024.1423320

**Published:** 2024-09-18

**Authors:** Jiaxuan Wang, Qianqian Qu, Xianzhao Zheng, Xiaoli Ma, Wenhao Cui, Zheng Lv, Cong Hu, Shiyao Li, Jiongbo Zhao, Haidong Lv

**Affiliations:** Department of Neurology, Jiaozuo People's Hospital of Xinxiang Medical University, Jiaozuo, China

**Keywords:** Andersen-Tawil syndrome, KCNJ2 gene, ion channelopathies, periodic paralysis, developmental malformations, myopathology

## Abstract

**Purpose:**

To explore the clinical, muscle pathological, and pathogenic gene mutation characteristics of Andersen-Tawil Syndrome (ATS) and enhance the understanding of ATS among clinical practitioners.

**Methods:**

Retrospective analysis of clinical data and muscle pathology of two ATS families, along with genetic testing for probands and some family members.

**Results:**

In Family 1, spanning four generations, four individuals were affected, while Family 2 had two affected individuals across four generations. All six patients in both families experienced onset in childhood, presenting with periodic paralysis, arrhythmias, and craniofacial skeletal abnormalities. In Family 1, the proband’s periodic paralysis was more triggered by low temperature and exercise, occurring several times a year, lasting 4–7 days. All three adult patients in Family 1 had a history of hypokalemia, and the frequency and severity of attacks were reduced after regular oral potassium supplement therapy. Two adult females in Family 1 experienced limb weakness triggered by stress, exertion, and premenstrual period, with milder symptoms than the proband. In Family 2, the proband’s periodic paralysis typically occurred the day after excessive exertion, with a frequency of approximately 2–3 months. Two years prior, the proband developed arrhythmias without palpitations or chest tightness. The proband’s brother experienced intermittent limb weakness during adolescence, remained untreated, and had sudden death at age 40. Physical examination revealed characteristic features in Family 1 and both probands: small mandible, wide eye spacing, and fifth-digit clinodactyly. Four adult patients were shorter in stature, while the growth status of a pediatric patient was indeterminate. Supplementary tests showed a history of hypokalemia during muscle weakness episodes in Family 1, while Family 2 patients had normal potassium levels during episodes. The long exercise tests were positive in both probands. Muscle MRI showed no significant abnormalities, but muscle pathology revealed rimmed vacuoles and tubular aggregates. Genetic testing identified KCNJ2 gene mutations in two probands and some of their family members, with c.407C > T (p.S136F) heterozygous mutation in Family 1 and c.652C > T (p.R218W) heterozygous mutation in Family 2.

**Conclusion:**

Among the clinical symptoms of the patients with Andersen-Tawil Syndrome in this study, not everyone exhibits the full triad of signs: periodic paralysis is the most common initial symptom, craniofacial and digit skeletal abnormalities are characteristic signs, and ventricular arrhythmias pose the most serious potential risk. Given that these typical symptoms were observed in 5 out of 6 patients, clinicians should pay special attention to these typical symptoms, and patients with these symptoms should be followed up over time. Muscle biopsy May reveal pathological changes such as tubular aggregates, but genetic testing for KCNJ gene mutations remains a crucial diagnostic criterion for this syndrome.

## 1 Introduction

Andersen-Tawil Syndrome (ATS) is a rare autosomal dominant inherited disorder characterized by a triad of features: periodic paralysis, ventricular arrhythmias, and craniofacial or digit skeletal abnormalities (1). Despite its dominant inheritance, the clinical phenotype of this condition exhibits high variability, resulting in a relatively low penetrance (2). Consequently, early clinical diagnosis poses significant challenges. Presently, the literature on ATS mostly comprises individual case reports, with limited documentation of family cases. This study retrospectively analyzes two ATS family members treated at the Jiaozuo People’s Hospital Neurology Department, exploring the clinical, muscle pathology, and genetic characteristics associated with ATS caused by KCNJ2 gene mutations. The aim is to enhance clinicians’ understanding and diagnostic proficiency in managing this condition.

This research received approval from the Jiaozuo People’s Hospital Ethics Committee, and all participants provided informed consent by signing an informed consent form.

## 2 Materials and methods

### 2.1 Study subjects

#### 2.1.1 Family 1

**Figure 1:**
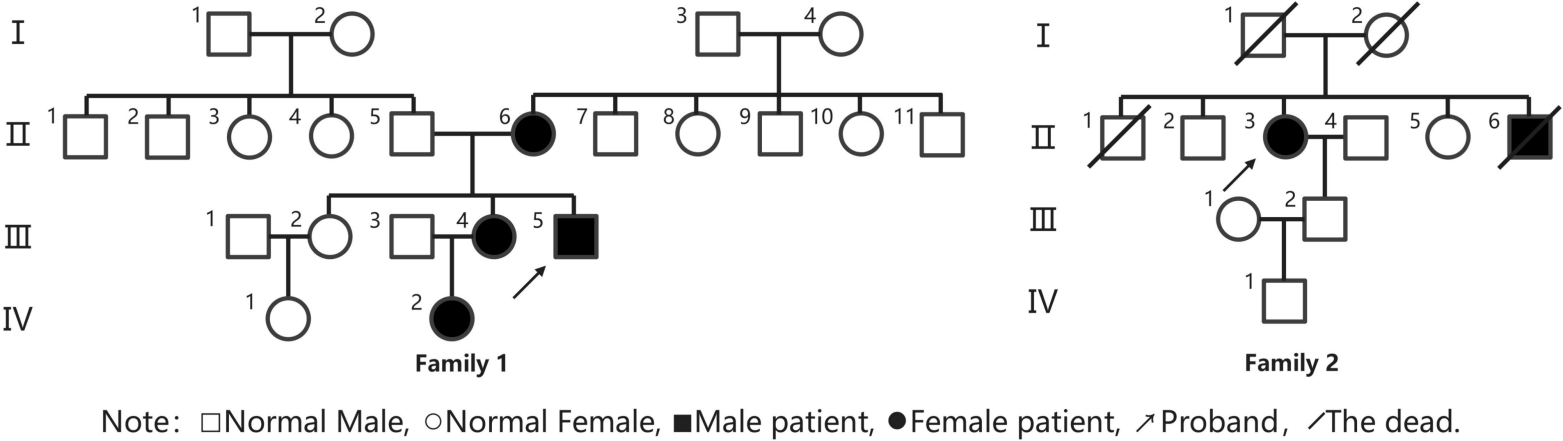
Family lineage diagram.

**Figure 2:**
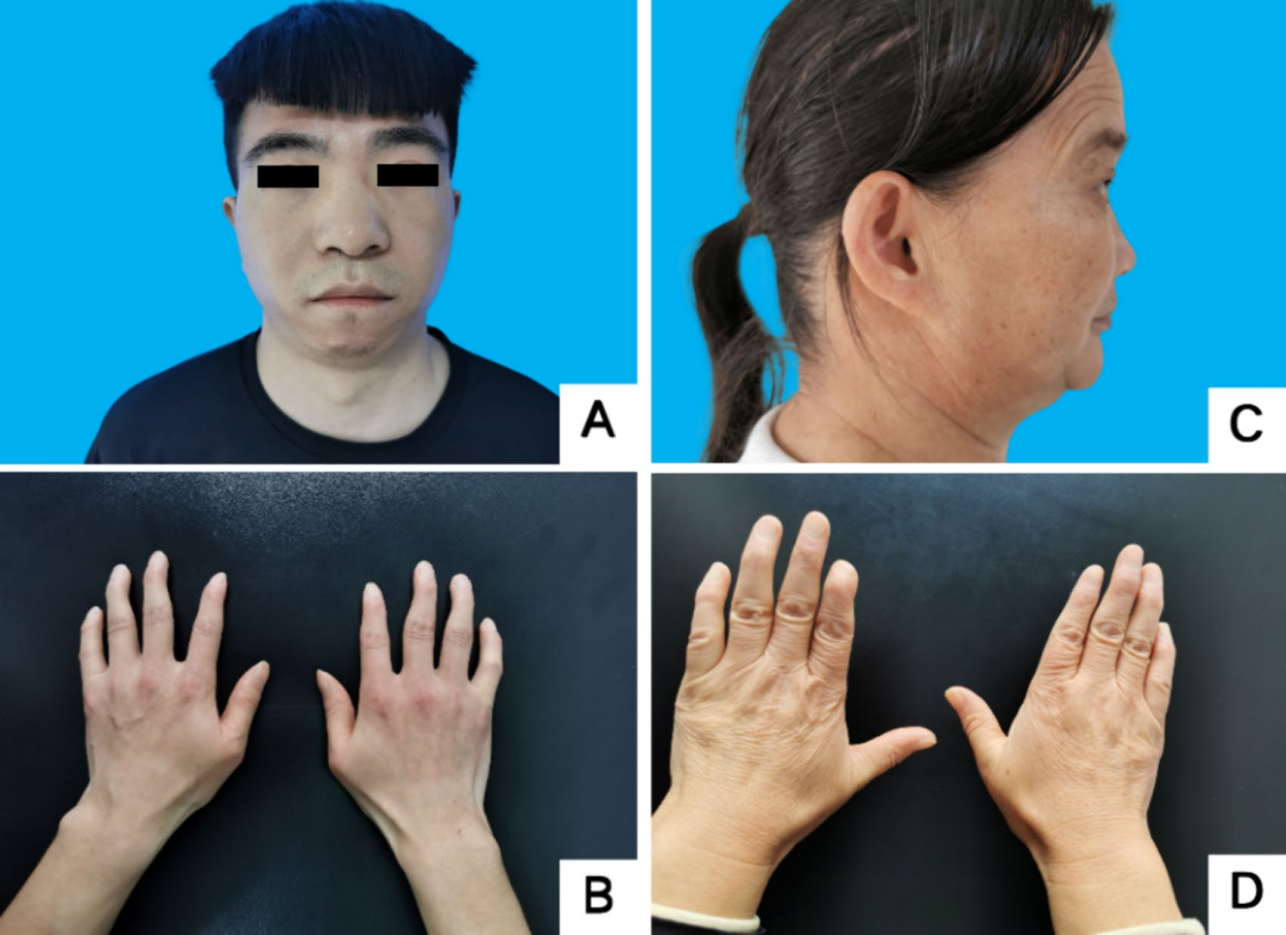
Facial and finger deformities of two probands. **(A,B)** Proband 1 III-5. **(C,D)** Proband 2 II-3. **A** showed wide eye spacing, broad nasal base and small mandible. **C** showed small mandible and low-set ears. **B,D** showed fifth-digit clinodactyly.

Proband III4, a 27-year-old male, presented at the Jiaozuo People’s Hospital Neurology Department in May 2016 with a 23-year history of “episodic limb weakness” (see Figure 1). He experienced episodic weakness in both lower limbs since the age of 4, making walking difficult, with difficulty standing up after squatting. The symptoms were often triggered by colds and excessive exercise. He had a history of low blood potassium, and regular oral potassium supplementation reduced the frequency and severity of attacks. After the age of 14, the patient’s symptoms worsened, the number of episodes increased from several times a year to more than a dozen, and the degree of weakness in the limbs during the episodes also worsened, and it was difficult to turn over and get up in severe cases. Two days after the attacks, the symptoms of muscle weakness began to gradually reduce, usually lasting a week. The weakness of both lower limbs was often felt between attacks, and it was not as good as people of the same age in walking up and down stairs and running. In the past 4 years, the patient had regular daily potassium supplementation, and the number of episodes was significantly reduced, and the weakness of the lower limbs was also reduced compared with the previous period. Physical Examination: The proband was 156 cm tall and weighed 51 kg. And he showed characteristic physical features like a small mandible, low-set ears, and wide eye spacing (see Figure 2A). Muscle strength was 5 in both upper limbs, 4–5 in the proximal muscle and 5 in the distal muscle of both lower limbs. Tendon reflexes in all four limbs were normal, and pathologic signs were negative. There were anomalies such as scoliosis and fifth-digit clinodactyly (see Figure 2B).

##### 2.1.1.1 Family history

The proband was born from non-consanguineous parents. The proband’s mother, 56 years old, also experienced intermittent lower limb weakness since the age of 7, often induced by stress, exertion, and premenstrual period. The episodes ranged from several to a dozen per year, with each lasting 3 to 5 days. The blood potassium was below normal in several laboratory tests, and the symptoms could be relieved after potassium supplementation. In the past 10 years, the number of episodes has been significantly reduced. The proband had two sisters, the eldest of whom had no similar symptoms. The second sister, 29 years old, had symptoms of limb weakness since the age of 6, with the lower limbs heavier than the upper limbs, and was bed-ridden in severe cases. And the weakness mostly induced by exertion and premenstrual period. Each time lasted 3 to 7 days, about 3 to 5 episodes per year, could be relieved after potassium supplementation therapy, and the condition has stabilized in the last 2 years with only one episode. The proband’s niece, 5 years old, experienced weakness in both lower limbs after having a cold and fever at the age of 4, which manifested as weakness in walking and difficulty standing up after squatting, and then the weakness was relieved after 3 days. The examination of the above three patients showed the presence of a small mandible, wide eye spacing, and fifth-digit clinodactyly. And their muscle tone and strength of limbs were normal.

#### 2.1.2 Family 2

Proband II3, a 49-year-old female, sought medical attention in September 2021 with a 30-year history of “episodic limb weakness” (see Figure 1). Symptoms began at the age of 13, while running in the morning, she developed pain and weakness in the lower limbs and was not able to run and walk normally, which lasted for 5 to 6 days, and gradually recovered. Afterwards, the attack occurred approximately every other month, the proband’s double lower limbs pain and weakness, mostly in the morning after waking up, and unable to walk normally, lasting 2 to 3 days could be completely relieved. In severe cases, she could not up and down the stairs and stand up after squatting, and had difficulty combing her hair and lifting her arms flat. The symptoms always lasted for about 1 week and completely returned to normal during remission. She had been to the local hospital, and her blood potassium and liver and kidney functions were normal, so she failed to make a definite diagnosis. In the past 10 years, the number of episodes was decreased and the symptoms of weakness were also reduced. One week ago, the limb weakness appeared again, and she could not stand up after squatting and lift both upper limbs flatly, so she came to our hospital. Physical examination: The proband was 153 cm tall and weighed 56 kg. She displayed characteristic physical features such as a small mandible, low-set ears, wide eye spacing and fifth-digit clinodactyly (see Figures 2C,D). Intelligence and speech were normal, and there were no cerebral nerve abnormalities. Neck flexor and extensor strength were normal. The muscle strength was 4 in the proximal muscle and grade 5 in the distal muscle of both upper limbs, grade 4 in the proximal muscle of both lower limbs, and the strength of the dorsiflexors and plantarflexors was 4 to 5. No significant muscle atrophy was observed, and both arches were normal.

##### 2.1.2.1 Family history

The proband’s parents were not closely related, and both deceased. There were four siblings, wherein the proband’s little brother had intermittent limb weakness as an adolescent, which was not formally diagnosed and treated, and died suddenly at the age of 40. The proband has one son in good health.

### 2.2 Methods

#### 2.2.1 Routine laboratory tests

Included blood and urine analysis, thyroid function, liver and kidney function, electrolytes, and muscle enzymes. Additional tests involved electrocardiography, echocardiography, hand and spine X-rays, lower limb muscle MRI, and neurophysiological examinations, including needle electromyography, nerve conduction, and long exercise test.

#### 2.2.2 Muscle pathology examination

After signing the informed consent form, probands from both families underwent quadriceps muscle pathological biopsy. Muscle specimens were fixed by isopentane freezing in isopentane cooled in liquid nitrogen, and sliced serial frozen sections. Sections were subjected to conventional histological, enzyme, and histochemical staining, including hematoxylin and eosin (H&E), modified Gomori trichrome (MGT), oil red O (ORO), periodic acid-Schiff (PAS), reduced nicotinamide adenine dinucleotide-tetrazolium reductase (NADH-TR), succinate dehydrogenase (SDH), cytochrome c oxidase (COX) and ATPase (pH4.5 and pH10.3). Results were visualized using a light microscope (magnifications, x200 and x400).

#### 2.2.3 Genetic testing

With informed consent, genetic testing was conducted on probands and selected family members. A 4 mL venous blood sample was used for genomic DNA extraction, followed by second-generation sequencing and family validation by a genetic testing company.

## 3 Results

### 3.1 Clinical features

In this study, the main clinical manifestation is episodic periodic paralysis, with lower limb weakness more prominent than upper limb weakness. As patients age, the frequency of episodes gradually decreases, with triggers such as exercise, fatigue, and premenstrual periods. The episodes occur several times to a dozen times per year, typically lasting 4 to 7 days and improving. In Family 1, three adult patients had a history of hypokalemia, and regular oral potassium supplementation could reduce the frequency and severity of episodes. The proband in Family 2 had no clear history of hypokalemia, and the frequency of attacks was less than that of Family 1. Physical examination revealed characteristic features in 5 patients from both families, including small mandible, wide eye spacing, and fifth-digit clinodactyly. Among them, 4 adult patients were relatively short in stature, while the status of a child patient in the growth and development period could not be determined. No cardiac involvement symptoms were observed in Family 1 members, and cardiac auxiliary examinations showed no significant abnormalities. The proband in Family 2 had frequent premature ventricular contractions and bigeminy on the electrocardiogram, and her brother experienced sudden death, suggesting severe arrhythmia as a possible cause.

### 3.2 Routine laboratory tests

**Figure 3:**
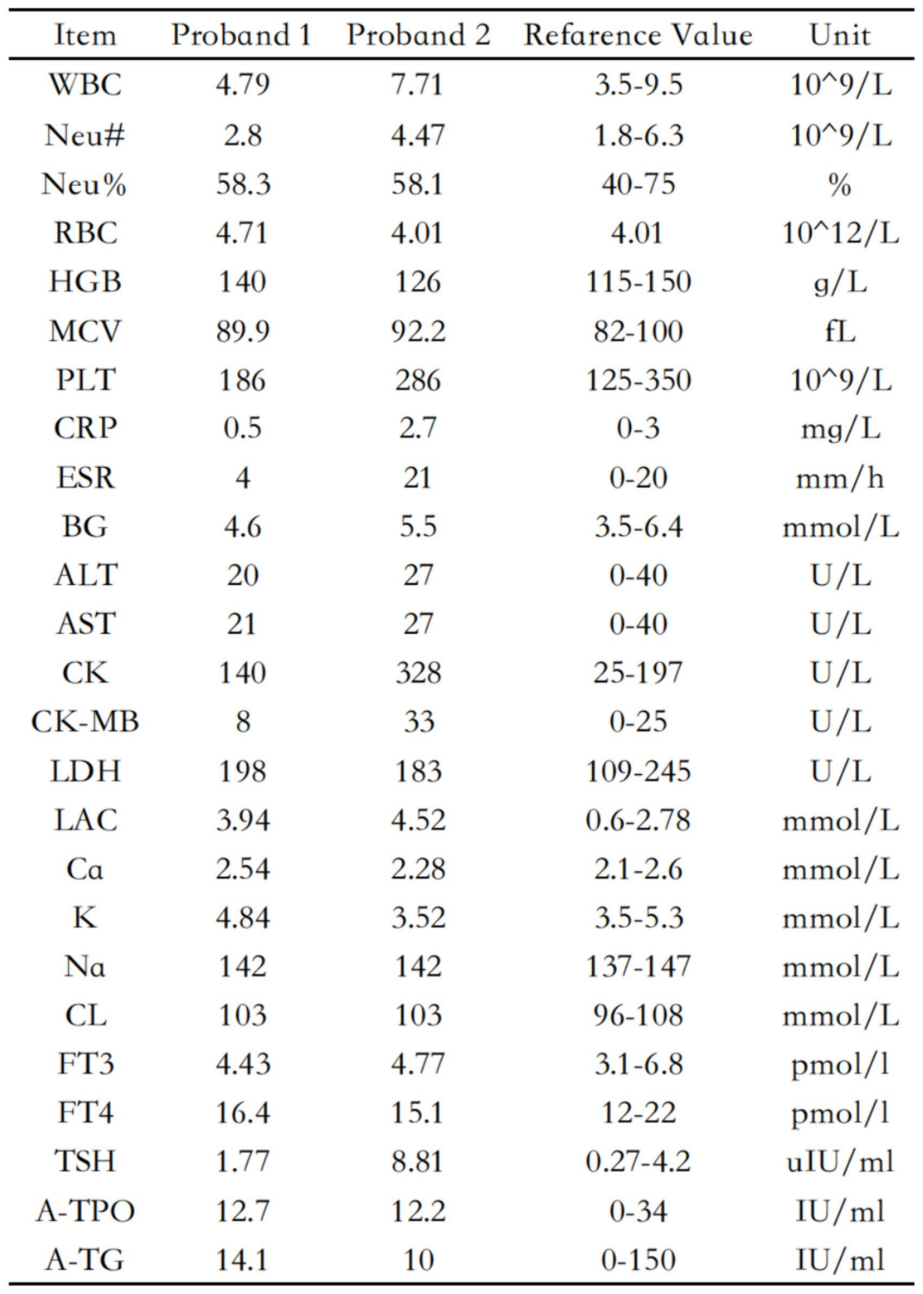
Laboratory data of two probands.

**Figure 4:**
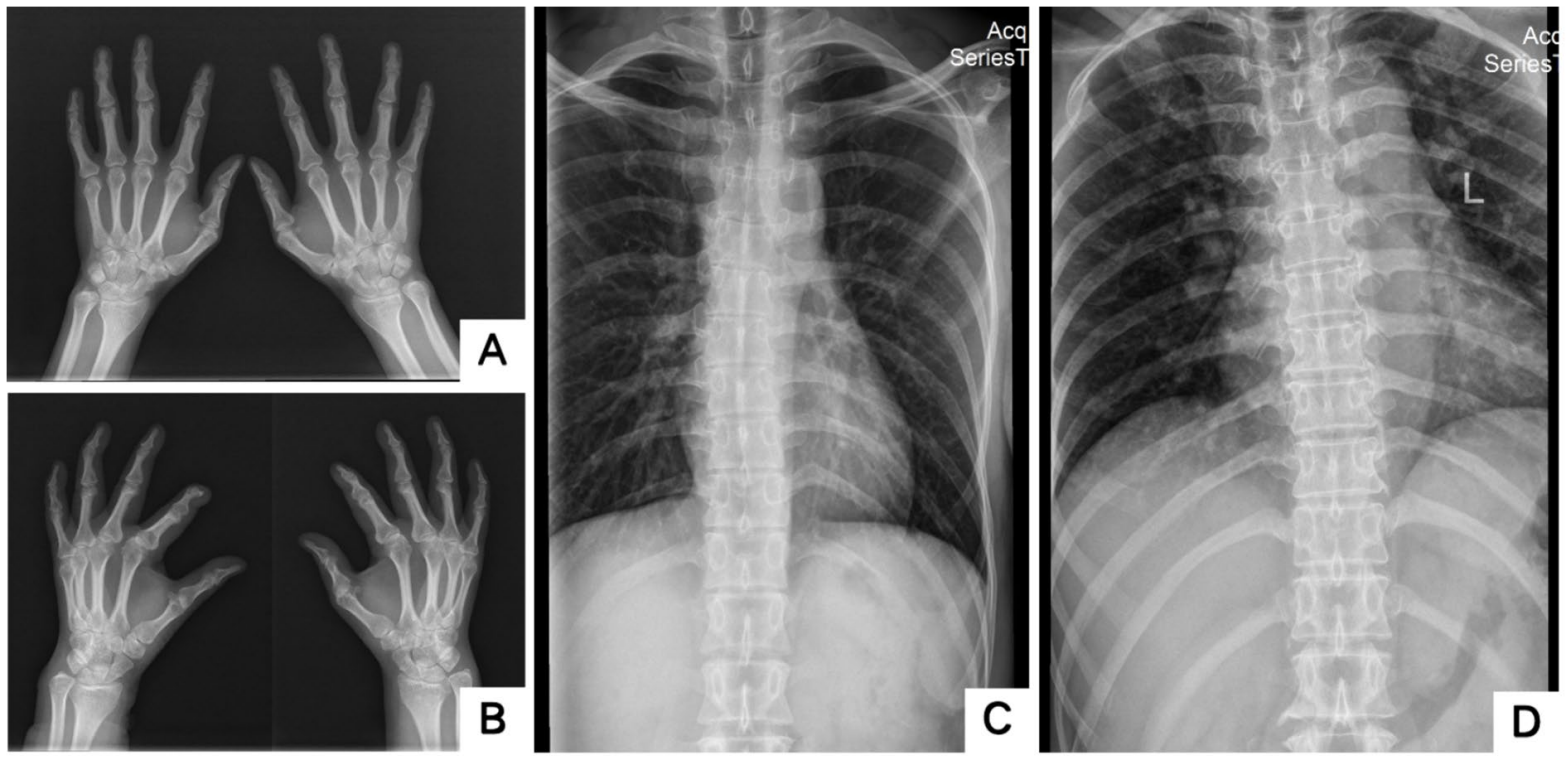
X-rays of two probands’ hands and scoliosis. **A** showed bilateral fifth-digit clinodactyly; **B** showed flexion deformity of the phalangeal joint of the left index finger; **C,D** showed Mild scoliosis (**A,C**: Proband 1 III-5; **B,D**: Proband 2 II-3).

**Figure 5:**
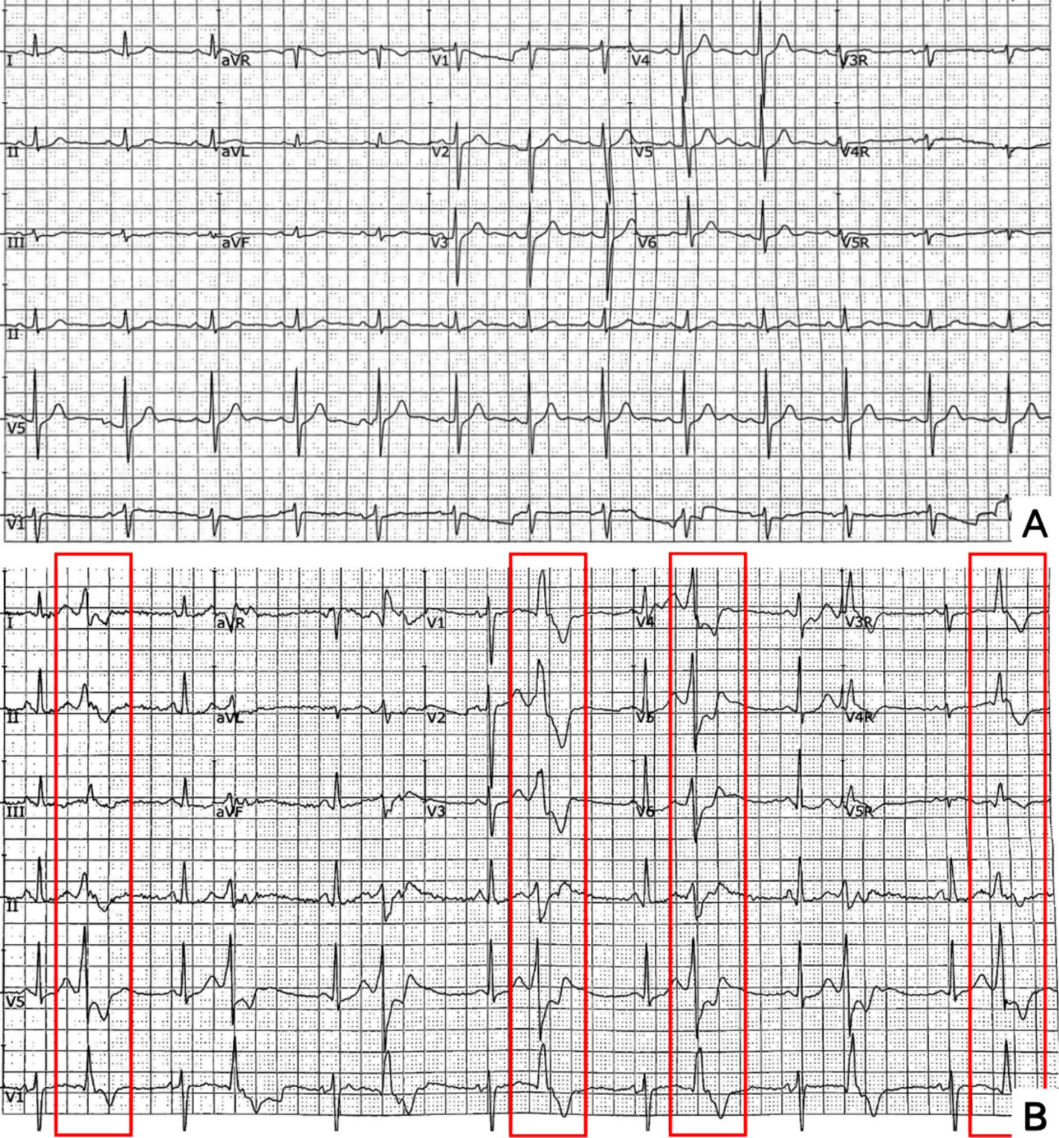
ECG. **A** (Proband 1 III-5) is normal. **B** (Proband 2 II-3) showed sinus rhythm and frequent premature ventricular beats with bigeminy (red rectangles).

**Figure 6:**
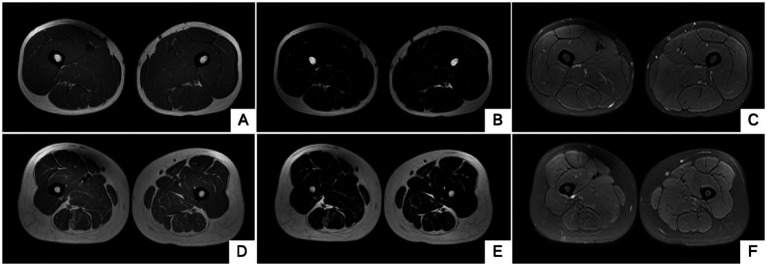
Muscle MRI of two probands’ thighs. No anomalies found. MRI of both thighs were taken at the level that is approximately 25 cm above the knee. **(A–C)** Proband 1 III-5. **(D–F)** Proband 2 II-3. **A**/**D**, **B**/**E** and **C**/**F** are T1WI, T2 water suppression and T2 fat suppression, respectively.

The blood routine, thyroid function, liver and kidney function, electrolytes, and muscle enzymes of 2 probands were all normal (see Figure 3). Family 1 members’ electrocardiogram showed no obvious abnormalities, and X-rays of both hands revealed bilateral fifth-digit clinodactyly (see Figure 4A). The proband’s electrocardiogram in Family 2 showed sinus rhythm, frequent premature ventricular contractions, and bigeminy (see Figure 5), and X-rays of both hands showed flexion deformity in the index finger joint of the left hand (see Figure 4B). The spinal X-ray of two probands revealed mild scoliosis (see Figures 4C,D). Echocardiography and muscle MRI showed no significant abnormalities (see Figure 6). Neurophysiological examinations of the 2 probands: Needle electromyography and nerve conduction were normal. The long exercise test showed a 58 and 65% reduction in left ulnar nerve CMAP amplitude after 10 min of exercise, indicating a positive result.

### 3.3 Muscle pathological features

**Figure 7:**
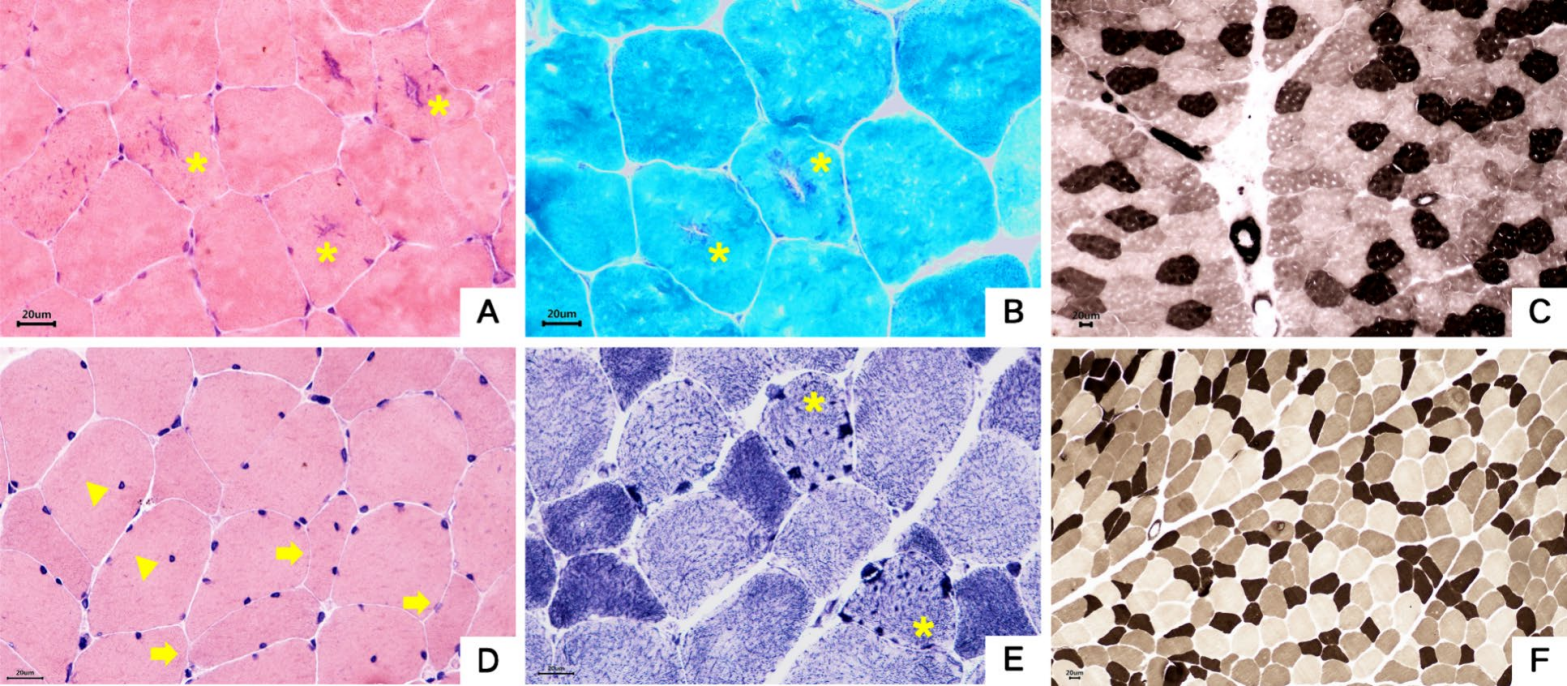
Muscle pathology. **(A–C)** Proband 1, III-5. **(A)** Muscle fibers were basically normal in size, some fibers had basophilic aggregates in the center, and some central aggregates had cleft-like changes (asterisk). HE staining × 400. **(B)** The central basophilic aggregates of myofibrils were partially red-stained (asterisk), and typical ragged-red fibers were not seen.GT staining × 400. **(C)** Type I and II fibers were distributed in a checkerboard, mosaic pattern, and no obvious grouping of fibers of the same type was seen. ATPase staining×200. **(D–F)** Proband 2, II-3. **(D)** The mildly varied size of muscle fiber diameters were diffusely distributed, a few round and strip-shaped atrophic muscle fibers (arrowhead) were seen, and a small number of muscle fibers showed splitting and intranuclear migration (triangle). HE staining × 400. **(E)** Deeply stained granular aggregates were seen in some muscle fibers (asterisk). NADH staining×400. **(F)** Type I and II fibers were distributed in a checkerboard, mosaic pattern, and no obvious grouping of fibers of the same type was seen. ATPase staining ×200.

Both probands underwent pathological biopsy of the quadriceps muscle. Muscle pathology revealed essentially normal-sized muscle fibers without obvious degeneration or necrosis. In Family 1 proband’s H&E staining, some muscle fibers showed rimmed vacuoles, and in Family 2 proband’s NADH-TR staining, some muscle fibers contained dark-staining granular material, indicating pathological evidence of tubular aggregates (see Figure 7).

### 3.4 Genetic testing results

**Figure 8:**
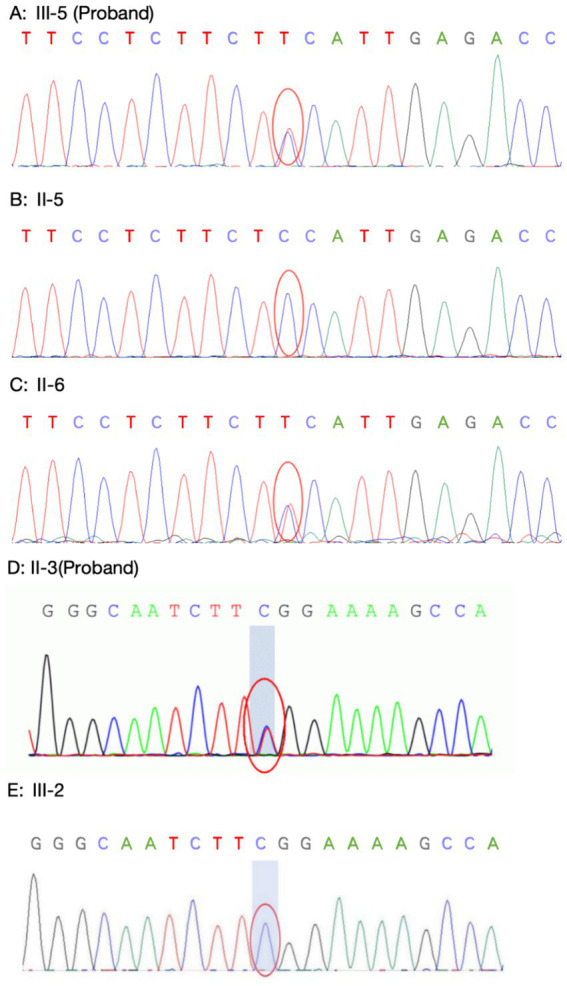
Sanger sequencing of KCNJ2 gene. (**A–C**: Family 1) Proband 1 **(A)** carried the same heterozygous c.407C > T (p.S136F) variant of the KCNJ2 gene as his mother **(B)**, and his father **(C)** did not carry this variant. (**D–E**: Family 2) The Proband 2 **(D)** carried a heterozygous c.652C > T (p.R218W) variant of the KCNJ2 gene, which was not carried by her son **(E)**.

Family 1 proband’s genetic testing result showed a heterozygous mutation in the KCNJ2 gene, c.407C > T (p.S136F). Family verification confirmed that the proband’s mother carried the same genetic mutation, while his father did not (see Figures 8A–C). Family 2 proband had a heterozygous mutation in the KCNJ2 gene, c.652C > T (p.R218W), and her son did not carry this genetic mutation (see Figures 8D,E). No mutations in the KCNJ5 gene or other pathogenic genes of ion channelopathy (CLCN1, SCN4A, CACNA1S, etc.) were found in any of the members who underwent genetic testing.

## 4 Discussion

In 1971, Andersen et al. (3) reported a patient with clinical manifestations of recurrent muscle weakness, premature ventricular beats, and facial developmental abnormalities, and suggested for the first time that a patient with the above triad of symptoms might be a specific syndrome. In 1994, Tawil et al. (4) retrospectively analyzed 10 patients with these clinical features, coining the term “Andersen syndrome” for the first time. In 2003, to acknowledge the contributions of both, the syndrome was named Andersen-Tawil syndrome (5).

Episodes of muscle weakness in ATS patients can present as hypokalemic, normokalemic or hyperkalemic periodic paralysis, with hypokalemic periodic paralysis being most common (1) Potassium supplementation can reduce the frequency of muscle weakness episodes and improve symptoms. Paralysis can be triggered by fatigue, emotional stress, or a high-carbohydrate diet (6). In this study, the probands in two families exhibited hypokalemic and normokalemic periodic paralysis, consistent with literature reports. We observed a gradual decrease in the frequency and severity of muscle weakness episodes with age in ATS patients.

Ventricular arrhythmias are one of the three major features of ATS and a significant factor leading to sudden death (7). Arrhythmias include frequent premature ventricular contractions, polymorphic ventricular tachycardia, and prominent U-waves and QT-interval prolongation (4). A reduction in inward rectifying potassium channels in Purkinje cells is suggested as a reason for increased susceptibility to ventricular arrhythmias in ATS patients (8). ATS patients with ventricular arrhythmias May be asymptomatic or present with palpitations; in severe cases, fainting, cardiac arrest, and sudden death can occur (7). The proband in Family 2 had typical frequent premature ventricular contractions and bigeminy but was asymptomatic. However, her brother experienced sudden cardiac death, likely due to severe ventricular arrhythmias. Therefore, it is recommended to perform echocardiography and 72-h Holter monitoring for all genetically confirmed ATS patients to detect potentially life-threatening arrhythmias early and provide proactive management and monitoring.

Developmental abnormalities are common in ATS, manifesting as a small mandible, wide eye spacing, low-set ears, fifth-digit clinodactyly, and scoliosis, among which a small mandible is the most characteristic manifestation (1, 9). Among the six patients in our two families, four exhibited short stature, and five showed typical signs of a small mandible, wide eye spacing, and fifth-digit clinodactyly, providing objective evidence for clinical diagnosis. Although these characteristic facial and finger skeletal developmental abnormalities May have mild clinical manifestations, they often serve as important clues for diagnosing ATS and should alert clinicians.

A relatively specific neurophysiological test for ATS is the long exercise test, which involves using bipolar electrodes to stimulate the ulnar nerve with a large stimulus and recording compound muscle action potential (CMAP) of the abductor digiti minimi muscle. If, after 20–40 min, CMAP amplitude drops by more than 40% from peak or the area decreases by more than 50%, it indicates ion channel abnormalities (1, 10). Both probands in our study had positive long exercise tests, showing a drop in CMAP amplitude by over 50% within 10 min after exercise. This result aligns with the conclusion that CMAP amplitude decreases earlier in ATS (11).

Typical muscle pathological changes in ion channelopathies include vacuoles and tubular aggregates appearing beneath the sarcolemma or within muscle fibers, possibly due to abnormal sarcoplasmic reticulum expansion and protein deposition; tubular aggregates are more common in ATS patients (12). A study evaluated 69 ATS patients with neuromuscular assessments (13), and 10 patients underwent lower limb muscle MRI, revealing fat accumulation in the posterior thigh and calf in seven patients. Muscle biopsies from five patients showed tubular aggregates in two. Although both probands in our study had no clear abnormalities on bilateral thigh MRI, muscle pathology revealed rimmed vacuoles and tubular aggregates. While the presence of rimmed vacuoles and tubular aggregates aids in diagnosing ATS, it lacks specificity; therefore, genetic testing remains a crucial diagnostic tool.

ATS is inherited in an autosomal dominant manner and is classified into ATS1 and ATS2 based on the causative gene. ATS1 caused by mutations in the KCNJ2 gene accounts for the majority of ATS cases, and the exact percentage requires further study in the future (1). The KCNJ2 gene encodes the inward rectifier potassium channel Kir2.1, expressed mainly in the heart, brain, and skeletal muscles (6, 14). Mutations in KCNJ2 lead to structural and functional impairment of the potassium channel, preventing its normal insertion into the cell membrane. This results in abnormal potassium transport in skeletal and cardiac muscle cells, ultimately causing periodic paralysis and arrhythmias (7). ATS2 is relatively rare and caused by mutations in the KCNJ5 gene and other sporadic mutations. Genetic testing in the probands of both families revealed KCNJ2 gene mutations: the proband in Family 1 had a c.407C > T (p.S136F) heterozygous mutation, inherited from the mother, and the proband in Family 2 had a c.652C > T (p.R218W) heterozygous mutation. The HGMDpro database reported that the variant loci c.407C > T and c.652C > T were both pathogenic variants (14, 15).

In summary, periodic paralysis, ventricular arrhythmias, and developmental abnormalities constitute the typical triad of Andersen-Tawil syndrome. In this study, periodic paralysis is the most common initial symptom, craniofacial and digit skeletal abnormalities are characteristic signs, and ventricular arrhythmias pose the most serious potential risk. Given that these typical symptoms were observed in 5 out of 6 patients, clinicians should pay special attention to these typical symptoms, and patients with these symptoms should be followed up over time. Some ATS patients May exhibit muscle pathology with tubular aggregates, and genetic testing, especially for KCNJ gene mutations, is crucial for definitive diagnosis.

## Data Availability

The datasets presented in this study can be found in online repositories. The names of the repository/repositories and accession number (s) can be found in the article/supplementary material.
